# Draft Genome Sequence of *Haloparvum sedimenti* Strain DYS4, the Type Species of the Genus *Haloparvum*, Isolated from a Salt Mine

**DOI:** 10.1128/genomeA.00770-17

**Published:** 2017-12-07

**Authors:** Shaoxing Chen, Chuanming Wang, He Li, Yunmei Shen

**Affiliations:** a College of Life Sciences, Anhui Normal University, Wuhu, Anhui, China; b Department of Biology, School of Life Science and Technology, Honghe University, Mengzi, Yunnan, China

## Abstract

Here is the genome sequence of *Haloparvum sedimenti* DYS4, the type species of the genus *Haloparvum*, isolated from a salt mine. The DNA G+C content of this genome was 68.27 mol%. The scaffold *N*_50_ was 96,635 bp. The completely sequenced and annotated genome is 3,243,052 bp and contains 3,313 genes.

## GENOME ANNOUNCEMENT

The solid ecosystem, for instance, a salt mine, blocks gene flow, which promotes the occurrence of speciation. Thus, the subterranean salt mine could be taken as a big pool of species and gene resources. The sample used for strain isolation was taken from a salt mine hundreds of meters underground, which is rich in halophilic microbes (1). Strain DYS4 (= CGMCC 1.14998^T^ = JCM 30891^T^) was isolated from a salt mine sample from Jiangcheng Salt Mine, Yunnan, China (101°38′33″E, 22°40′50″N). According to the minimal standards for the description of new taxa in the order *Halobacteriales* proposed by Oren et al. (2), a new genus, *Haloparvum*, was established (3). At the writing of this paper, this genus contains two species, *Haloparvum sedimenti* (3) and *Haloparvum alkalitolerans* (4). *Haloparvum sedimenti* is assigned the type species of this genus. Based on the phylogenomic analyses, the class *Halobacteria* has three orders, *Halobacteriales*, *Haloferacales*, and *Natrialbales* (5). The genus *Haloparvum* should be placed into the family *Halorubraceae* (order *Haloferacales*) (6).

Strain DYS4 was isolated by using the medium AS-168 (pH 8.0), which contains 200 g/liter NaCl, 20 g/liter MgSO_4_·7H_2_O, 5 g/liter yeast extract (Difco), 5 g/liter Casamino Acids (Difco), 3 g/liter sodium citrate, 2 g/liter KCl, 1.8 g/liter monosodium glutamate, 0.036 g/liter FeSO_4_, and 0.0036 MnCl_2_·4H_2_O (7). The total DNA was isolated and purified according to the methods described by Marmur (8). The NEBNext Ultra II DNA library prep kit for Illumina was used to create libraries for genome sequencing, which was performed on the Illumina HiSeq 2500 platform after paired-end library construction with a 500-bp insert size at the Beijing BioMarker Sequencing Center.

*De novo* assembly of short reads into contigs was performed using Velvet (9), with 207× genome coverage. Contigs <1,000 bp were deleted. A total of 143 scaffolds yielded a genome sequence 3,243,052 bp long, with a G+C content of 68.27%. Open reading frame (ORF) prediction and automatic annotation were performed using the NCBI Prokaryotic Genome Annotation Pipeline (http://www.ncbi.nlm.nih.gov/genomes/static/Pipeline.html) and the software Glimmer (interpolated Markov models [IMMs]) (10). The complete genome sequence contains 3,313 genes, 3,074 coding sequences (CDSs), 9 microRNAs, 2 rRNAs (16S), and 35 tRNAs.

Five possible prophages were detected on the genome by using the software PHAST (11); however, only two of them are located at the classical insertion site of the tRNA. Furthermore, five typical clustered regularly interspaced short palindromic repeat (CRISPR) structures were detected from scaffold_27, scaffold_62, scaffold_105, and scaffold_142 by using the online software CRISPRFinder (http://crispr.i2bc.paris-saclay.fr/). Additionally, seven pseudogenes were detected using the software GeneWise (12). These bioinformatic analyses hint that this strain could be taken as a good example for studying the defense system of haloarchaea.

According to phylogenetic analysis, the strain *Haloparvum sedimenti* DYS4 is most closely related to species of the genera *Halorubrum* and *Halopenuts*.

### Accession number(s).

The draft genome sequence for *Haloparvum sedimenti* strain DYS4 has been deposited in GenBank under the accession number LKIR00000000 (BioProject number PRJNA294239, BioSample number SAMN04018174). The 56 scaffolds have been deposited under the accession numbers LKIR01000001 to LKIR01000043. The version described in this paper is the first version.
